# Combining Robotic Training and Non-Invasive Brain Stimulation in Severe Upper Limb-Impaired Chronic Stroke Patients

**DOI:** 10.3389/fnins.2016.00088

**Published:** 2016-03-08

**Authors:** Vincenzo Di Lazzaro, Fioravante Capone, Giovanni Di Pino, Giovanni Pellegrino, Lucia Florio, Loredana Zollo, Davide Simonetti, Federico Ranieri, Nicoletta Brunelli, Marzia Corbetto, Sandra Miccinilli, Marco Bravi, Stefano Milighetti, Eugenio Guglielmelli, Silvia Sterzi

**Affiliations:** ^1^Unit of Neurology, Neurophysiology, Neurobiology, Department of Medicine, Università Campus Bio-Medico di RomaRome, Italy; ^2^Fondazione Alberto Sordi - Research Institute for AgeingRome, Italy; ^3^Unit of Biomedical Robotics and Biomicrosystems, Department of Engineering, Università Campus Bio-Medico di RomaRome, Italy; ^4^Unit of Physical and Rehabilitation Medicine, Department of Medicine, Università Campus Bio-Medico di RomaRome, Italy

**Keywords:** stroke recovery, robot-assisted rehabilitation, non-invasive brain stimulation, homeostatic plasticity, robotic assessment of motor performance

## Abstract

Previous studies suggested that both robot-assisted rehabilitation and non-invasive brain stimulation can produce a slight improvement in severe chronic stroke patients. It is still unknown whether their combination can produce synergistic and more consistent improvements. Safety and efficacy of this combination has been assessed within a proof-of-principle, double-blinded, semi-randomized, sham-controlled trial. Inhibitory continuous Theta Burst Stimulation (cTBS) was delivered on the affected hemisphere, in order to improve the response to the following robot-assisted therapy via a homeostatic increase of learning capacity. Twenty severe upper limb-impaired chronic stroke patients were randomized to robot-assisted therapy associated with real or sham cTBS, delivered for 10 working days. Eight real and nine sham patients completed the study. Change in Fugl-Meyer was chosen as primary outcome, while changes in several quantitative indicators of motor performance extracted by the robot as secondary outcomes. The treatment was well-tolerated by the patients and there were no adverse events. All patients achieved a small, but significant, Fugl-Meyer improvement (about 5%). The difference between the real and the sham cTBS groups was not significant. Among several secondary end points, only the Success Rate (percentage of targets reached by the patient) improved more in the *real* than in the *sham* cTBS group. This study shows that a short intensive robot-assisted rehabilitation produces a slight improvement in severe upper-limb impaired, even years after the stroke. The association with homeostatic metaplasticity-promoting non-invasive brain stimulation does not augment the clinical gain in patients with severe stroke.

## Introduction

Severe upper limb impairment in chronic stroke patients does not respond to standard rehabilitation strategies; for this reason there is the need of new treatments that might be effective in patients with drastically limited residual movement capacity. In patients with moderate to severe upper-limb impairment, a slight improvement have been reported using robot-assisted rehabilitative treatment, even years after a stroke (Lo et al., 2010). Another innovative approach for the enhancement of motor recovery is represented by non-invasive human brain stimulation techniques, such as repetitive transcranial magnetic stimulation (rTMS) and transcranial direct current stimulation (tDCS). These techniques can induce long-lasting changes in the excitability of central motor circuits via long-term potentiation/depression (LTP/LTD)-like phenomena (Di Pino et al., 2014b). A recent study reported a mild motor improvement after 10 sessions of rTMS in a group of severe chronic stroke patients (Demirtas-Tatlidedea et al., 2015).

Aim of present study was to explore whether the combination of these two approaches might enhance their positive effects on motor recovery. To the end of assessing safety and potential efficacy of the combination of robot-assisted rehabilitation and non-invasive brain stimulation in a group of chronic stroke patients with severe upper limb impairment, we designed a proof-of-principle double blinded semi-randomized sham-controlled trial. We used continuous theta burst stimulation (cTBS), a robust form of inhibitory rTMS inducing LTD-like changes lasting for about 1 h [8]. The choice of employing cTBS on the affected hemisphere was based on the findings of our recent study, which suggested that this inhibitory protocol can improve the response to physical therapy (Di Lazzaro et al., 2013). Moreover, rTMS protocols suppressing cortical excitability have been shown to strongly facilitate motor learning in normal subjects (Jung and Ziemann, 2009). Jung and Ziemann suggested that such enhancement might involve the phenomenon of “homeostatic” plasticity, which can be induced in the human brain using a variety of brain stimulation protocols (Karabanov et al., 2015). Considering the close link between LTP and mammalian learning and memory (Malenka and Bear, 2004), an enhancement of learning after LTD induction might appear a paradox. However, the experimental studies by Rioult-Pedotti et al. demonstrated the existence of a homeostatic balance between learning and the induction of LTP/LTD (Rioult-Pedotti et al., 2000), thus showing that the ease of producing synaptic LTP/LTD depends on the prior history of neural activity. In the context of stroke, this predicts that by delivering a rTMS protocol that induces LTD-like effects on the stroke-affected hemisphere before performing rehabilitation, would luckily result in better relearning (Di Pino et al., 2014a).

## Materials and methods

### Subjects

The study was performed according to the Oviedo Convention and approved by the Ethics Committee of Università Campus Bio-Medico of Rome. Participants provided written informed consent. Inclusion criteria were: (a) first-ever ischemic stroke at least 1 year earlier; (b) severe hand function impairment, defined as score of 3–28 on the Fugl-Meyer Assessment of sensory motor recovery after stroke, a scale with scores for upper-limb impairment ranging from 0 (no function) to 66 (normal function); (c) ability to give informed consent and comprehend instructions. Exclusion criteria were: (a) concomitant neurological conditions, including any history of epilepsy and significant comorbidities; (b) cognitive impairment or any substantial decrease in alertness, language reception, or attention that might interfere with understanding instructions for motor testing; (c) apraxia; (d) excessive pain in any joint of the paretic extremity; (e) contraindications to TMS such as metal head implants; (f) advanced liver, kidney, cardiac or pulmonary disease; (g) history of significant alcohol or drug abuse; (h) depression or use of neuropsychotropic drugs such as antidepressants or benzodiazepines. The National Institute of Health Stroke Scale (NIHSS) and the Barthel Index (BI) were used to evaluate neurological impairment and disability at the enrolment.

**Figure 1:**
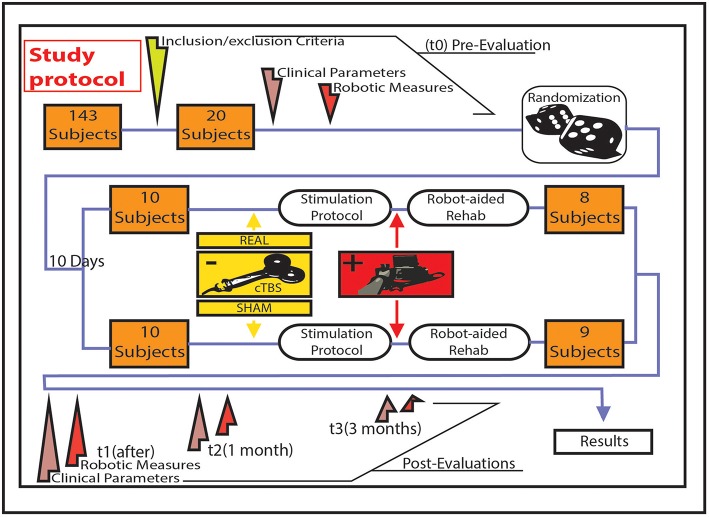
**Figurative illustration representing the algorithm of the study design, the evaluations carried out, and the treatments delivered**. Treatment (real/sham cTBS + physical therapy) was delivered for 10 consecutive working days. Baseline evaluation was performed in the first day of treatment.

The study was proposed to patients attending the outpatient clinic for cerebrovascular disorders of Campus Bio-Medico University Hospital. From April the first, 2013, to September the 30th, 2014, we screened 143 patients, 13 of whom declined, 110 were excluded, and 20 underwent randomization (Figure 1). Common reasons for exclusion of patients from the study were a baseline Fugl-Meyer score outside the required range, history of epilepsy, and haemorrhagic stroke. Other causes of exclusion were previous ischemic strokes, stroke occurring < 1 year before, severe cognitive impairment, contraindications to TMS such as metal head implants or pacemaker, use of neuropsychotropic drugs such as antidepressants or benzodiazepines.

### Experimental design

Ten patients were randomized to robot-assisted therapy associated with real cTBS and 10 patients to robot-assisted therapy associated with sham cTBS, through a randomization stratification approach. Patients were stratified by using at baseline measures to ensure that both groups had a similar distribution regarding degree of impairment. Researcher randomizing patients and researchers delivering cTBS were not involved in outcome assessments and data analysis; rehabilitation doctors, patients, and researchers involved in data analysis were blind to the type of cTBS delivered (i.e., sham or real), in order to obtain a double-blinded sham-controlled study design.

Each day, for 10 consecutive working days, each patient received a session of robotic therapy following the real or sham stimulation. Patients were evaluated at four time points: baseline (Baseline), just after the treatment (Post), after 1 (1 Month), and 3 months (3 Months). For all these evaluation points we assessed the Fugl-Meyer score and Robotic measures of motor performance (Figure 1). At baseline we also included the following scales: NIHSS, Rankin Scale, Barthel Index, and Modified Ashworth Scale (MAS). Spasticity was assessed by MAS at four different joint of affected arm: shoulder, elbow, wrist, and fingers. For each patient, a cumulative score was obtained by summing the scores obtained in the four joints. The cumulative score ranges from 0 (no spasticity) to 16 (maximum spasticity, i.e., score 4 in all the considered joints).

The combined effect of robotic rehabilitation and brain stimulation was evaluated on (a) the Fugl Meyer score after intervention, as compared to baseline (primary outcome measure of clinical improvement) and (b) robot derived measures of motor performance (secondary outcome measures).

After the 2 weeks of intervention, patients did not receive any additional physical therapy until the last follow-up visit (at 3 months). Pharmacological therapy was also unchanged.

### Interventions

#### Transcranial brain stimulation

rTMS was applied over the hand motor area of the affected hemisphere using a DUOMAG XT stimulator (DEYMED Diagnostic, Czech Republic) and a figure-of-eight shaped coil, with the handle pointed posteriorly and approximately perpendicular to the central sulcus.

Active rTMS used cTBS, in which 3 pulses are given at 50 Hz, repeated every 200 ms for a total of 600 pulses. Stimulation intensity was 80% active motor threshold (AMT) of the affected hemisphere, defined as the minimum single pulse intensity required to produce a motor evoked potential >200 μV on more than 5 out of 10 trials from the contracted contralateral first dorsal interosseous muscle. Whenever AMT over the affected hemisphere could not be determined because TMS at maximum stimulator output (MSO) failed to evoke any response, cTBS intensity was performed at an intensity corresponding to unaffected hemisphere AMT. Sham rTMS was performed using the same stimulator at an intensity of 3% of MSO and with the coil tilted at 90°; this intensity of stimulation, with this orientation of the coil, produces auditory sensation similar to the active stimulation, but has no stimulating effect on the cortex.

#### Robotic therapy

**Figure 2:**
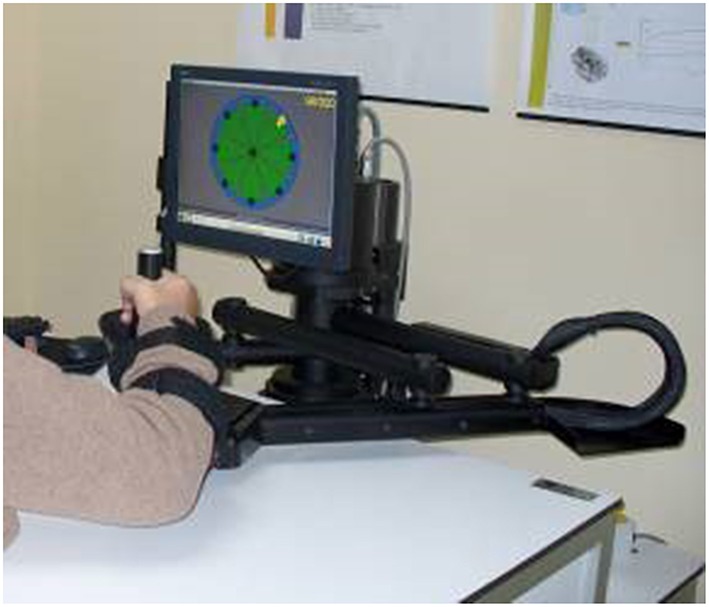
**The InMotion2 robotic machine (Interactive Motion Technologies, Inc.)**.

The Robot was exploited for the two-fold purpose of delivering therapy and measuring, objectively and quantitatively, patients' motor performance. Shoulder-elbow robotic therapy was delivered with the InMotion2 robotic machine (Interactive Motion Technologies, Inc.) (Krebs et al., 1998). The InMotion2 (Figure 2) is based on a direct-drive five-bar-linkage SCARA mechanism that provides two translational degrees of freedom for elbow and forearm motion. Impedance control enables the robot to move, guide or perturb the patient's movement. Absolute encoders at each motor and a 6-axis force/torque sensor at the end effector allow measuring robot joint position, robot Cartesian position (via forward kinematics) and interaction forces.

In the evaluation phase, the robot was completely passive while position sensors recorded subject kinematic data. Patients were asked to perform five blocks of unassisted 16 point-to-point movements from the center to eight outbound targets along a circle at a distance of 0.14 m. Patients were required to move with a self-paced speed in a maximum time slot of 3 s.

Robot data were offline processed to compute quantitative indicators of temporal and spatial features of motor skill recovery (Zollo et al., 2011a; Papaleo et al., 2013), i.e.:

***Motion Accuracy—***It is assessed by means of the area index and the normalized mean deviation, defined below:

- *AREA*. It is the area between the desired and the actual trajectory performed by the patient in the XY plane during the point-to-point motion; it is expected to decrease as movement accuracy increases with recovery.
- *normalized Mean Deviation (nMD)* (Colombo et al., 2008). It is the mean absolute value of the distance between the desired path and the curve actually performed by the patient, normalized on the maximum deviation (or on the length of the theoretical path). As the patient recovers, the deviation from the desired path is expected to decrease;

***Motion Direction—*** It is assessed through the *aiming angle*, i.e., the angular difference between the target direction and the direction of the path performed from the starting point up to peak speed point. It is expected to decrease as movement direction improves during recovery;

***Smoothness*** (Rohrer et al., 2002)—It is a measure of how gradually a movement is changing and it is characterized by peaks and deep valleys in the velocity profile. Smoothness is quantified through the indicators reported below:

- *Speed Metric (SM)*. It is expressed as the ratio between mean speed and peak speed. As patient recovers, the normalized mean velocity increases due to the reduction of peaks and valleys in the velocity profile;
- *Mean Arrest Period Ratio (MAPR)*. It represents the amount of time (i.e., the percentage of samples) that the movement speed exceeds the 10% of the peak speed. The deep valleys (percentage of pauses during the task execution) in the velocity profile of the patient hand are expected to reduce as movement smoothness improves.

***Speed*** —It quantifies the movement velocity by measuring the Deviation from Ratio between Velocities (DRV), defined as the absolute deviation of the ratio between peak velocity and mean velocity from the constant value 1.875 (corresponding to the value obtained in the minimum jerk trajectory) (Flash and Hogan, 1985). It is expected to reduce when patient velocity tends to the bell-shaped velocity profile of the minimum jerk trajectory.

***Movement Duration*** (MD)—It gives a measure of the task execution time, evaluated as the time occurred for performing a point-to-point movement from movement onset to movement termination. Movement onset is defined as the time instant where speed exceeds a predefined threshold of 10% of peak velocity and movement termination is defined as the time instant where velocity goes below a predefined threshold of 10% of peak velocity. As patient recovers, movement duration is expected to decreases as a consequence of the improved efficiency.

***Efficiency***—It evaluated the measure of patient ability to reach the target during point-to-point movement; it can be assessed by means of the path length index and the percentage of successes:

- *Path Length (PL)*. It is defined as the length ratio between the actual patient curve and the desired straight line, and computed as the line integral of the trajectory over the Movement Duration (MD), normalized with respect to the desired path. It is expected that during recovery the actual patient curve tends to the desired path and, hence, their ratio tends to one;
- *% Successes (SR)* (Panarese et al., 2012). It represents the percentage of times that the patient reaches the target during a therapy session of point-to-point movements. The increase of the SR with recovery is expected.

Each day of robotic treatment consisted of three sessions of 320 assisted point-to-point movements, from the center to eight outbound targets, interspersed by four sessions of 16 unassisted recorded point-to-point movements. Robot assistance at each session was tuned on patients' performance during the 16 point-to-point sessions. Both during evaluations and during training, patients were required to move with a self-paced speed in a maximum time slot of 3 s. Robotic treatment was delivered daily for 10 consecutive working days. A physical and rehabilitation medicine doctor attended and assisted patients both during evaluations and treatment.

### Statistics

Statistical analysis was performed using IBM SPSS v22. We verified that at baseline the two groups were matched regarding age, sex, and clinical status. Then we investigated the effect of brain stimulation and robotic rehabilitation on the primary outcome measure, namely Fugl-Meyer scores, using an ANOVA mixed model design, with *Time* (four levels: *Baseline, Post, 1 Month, 3 Months*) as within subject factor and *Group* (two levels: *real cTBS* and *sham cTBS*) as between subjects factor. For the secondary outcome measures (robot derived measures) we applied a Generalized Estimating Equation approach, as multiple values were available for each cell of the design (Pellegrino et al., 2012; Di Lazzaro et al., 2014). The autoregressive (lag = 1) working correlation within subjects was chosen because measures of motor performance were acquired consecutively. The study of the *Success Rate* was performed by means of the Chi-Square test. The level of significance was set at *p* < 0.05 and the alpha inflation due to multiple comparisons was faced according to Bonferroni's procedure whenever required. Descriptive statistics is reported as Mean ± Standard Error of the Mean (SE).

## Results

Twenty patients underwent randomization (14% of the screened patients): 10 to robot-assisted therapy associated with real cTBS and 10 to robot-assisted therapy associated with sham cTBS. One real patient withdrew consent before the first session of treatment. One real patient and 1 sham patient withdrew because of difficulty in reaching the hospital after the third and after the fifth day of treatment, respectively. Data of these patients was not included in the analysis. A total of 17 patients completed the study including the 3 month follow-up: 8 real cTBS patients (mean age: 57.8 ± 4.4 years) and 9 sham cTBS (mean age: 56.7 ± 3.2 years), therefore, 85% of the screened patients completed the study. For the purposes of the study we applied an on-treatment analysis (Figure 1). The real and sham groups were matched regarding age, sex, time elapsed from stroke onset, and clinical status at baseline (Table 1).

**Table 1:** **Demographic and clinical characteristics of the patients at baseline**.

	**Real cTBS (*n* = 8)**	**Sham cTBS (*n* = 9)**	***P*-value**
Age (years)	57.88 ± 4.434	56.78 ± 3.202	0.841
Sex (M)	4	4	1.000^a^
Months since stroke	63.25 ± 25.437	61.33 ± 14.716	0.541^b^
NIHSS	5.50 ± 0.779	5.00 ± 0.687	0.636^c^
Rankin	2.88 ± 0.350	3.00 ± 0.333	0.815^b^
Barthel index	76.88 ± 7.130	77.22 ± 4.648	0.743^b^
Modified ashworth scale cumulative score^*^	5.00 ± 0.597	7.111 ± 1.160	0.140^c^
Fugl-Meyer	14.50 ± 2.428	12.56 ± 2.243	0.565^c^

*All data are expressed as mean ± standard error*.

a *Chi-Square*.

b *Mann-Whitney*.

c *Two tailed independent sample t-test*.

* *Cumulative score was obtained by summing the scores obtained at four different joints of affected arm: shoulder, elbow, wrist, and fingers*.

Physicians inquired about adverse events and pain, each day during the whole stimulation period (10 consecutive working days) and at each outward control. There were no treatment-related adverse events. No patient reported pain in the affected arm subsequent to treatment or required to stop treatment session for pain or any other unpleasant sensation. In particular, patients reported no side effects that could be related either to the robotic treatment (e.g., shoulder, elbow, or wrist pain) or to cTBS (seizure, syncope, transient headache, local pain, neck pain, transient cognitive/neuropsychologial changes; Rossi et al., 2009).

### Primary outcome measure

**Figure 3:**
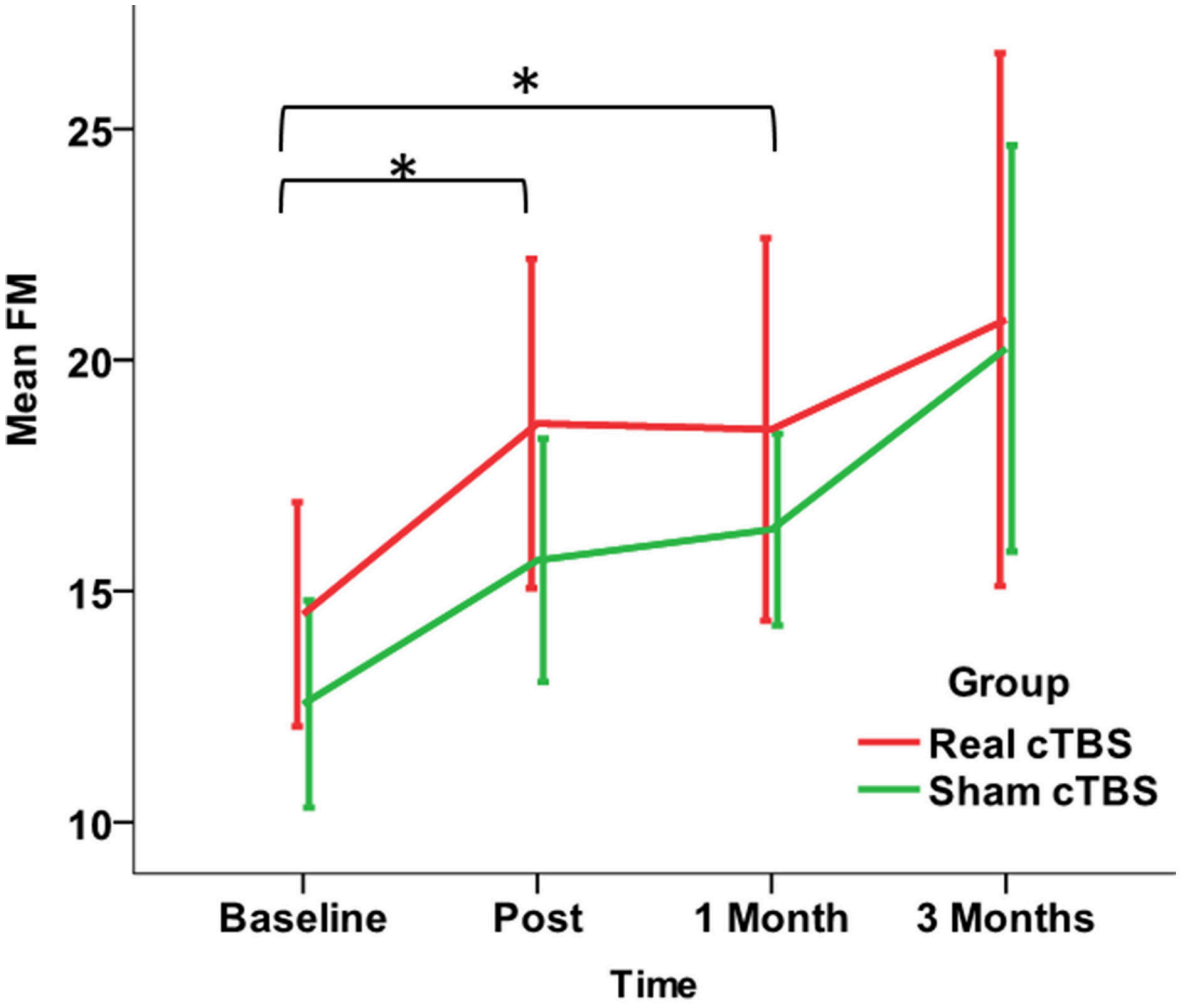
**Changes in the primary Outcome Measure (Fugl-Meyer Assessment score) in the *Real* (red line) and the *Sham* (green line) cTBS groups**. Compared to Baseline both groups significantly improved at t1 (post-treatment) and t2 (1 month). There is no significant difference between groups. ^*^p < 0.05.

The ANOVA Mixed Model with *Time* (four levels: *Baseline, Post, 1 Month, 3 Months*) as within subject factor and *Group* (two levels: *real cTBS* and *sham cTBS*) as between subjects factor revealed a significant effect of rehabilitation [*FactorTime*: *F*_(1.613, 22.586)_ = 5.801, *p* = 0.013], but no effect of the brain stimulation (*Factor Group* and *Group by Time interaction*: *p* > 0.200 consistently). The improvement vs. baseline was statistically significant both soon after the intervention (*Post*) and at *1 Month* follow-up (Bonferroni corrected *post-hoc p* = 0.30, *p* = 0.19, respectively). At *3 Months* there was an average additional increase of the Fugl-Meyer score, however the difference toward *Baseline* was not significant (Bonferroni corrected *post-hoc p* = 0.75) (Figure 3).

### Secondary outcome measures

#### Motion accuracy, motion direction, smoothness, speed, movement duration

**Figure 4:**
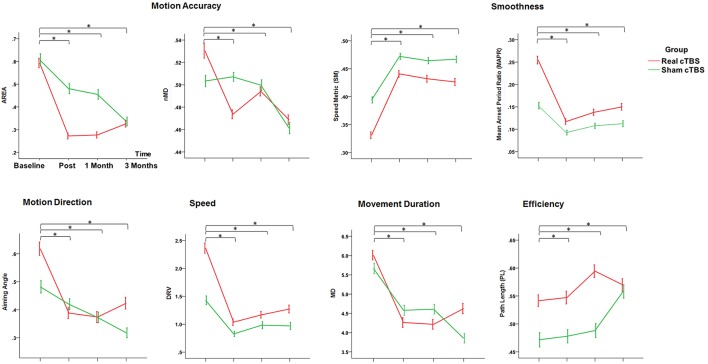
**Changes in the Secondary Outcome Measures (motor performance parameters extracted by the robot) in the *Real* (red line) and the *Sham* (green line) cTBS groups**. Compared to Baseline both groups significantly improved at t1 (post-treatment), t2 (1 month), and t3 (3 months) ^*^p < 0.05. There is no difference between groups.

The main finding was a rehabilitation-related improvement of the motor performance across multiple domains, including *Motion Accuracy, Motion Direction, Smoothness, Speed*, and *Movement Duration*. In particular all the robot-derived measures, except normalized Mean Deviation (nMD), consistently showed a significant factor *Time* (*Area*: Wald Chi-Square = 28.019, *df* = 3, *p* = 0.000; *Aiming angle (alpha)*: Wald Chi-Square = 44.608, *df* = 3, *p* = 0.000; *Speed Metric (SM):* Wald Chi-Square = 126.045, *df* = 3, *p* = 0.000; *Mean Arrest Period Ratio (MAPR)*:Wald Chi-Square = 2.796, *df* = 3, *p* = 0.000; *DRV*: Wald Chi-Square = 20.275, *df* = 3, *p* = 0.000; *Movement Duration (MD)*: Wald Chi-Square = 52.088, *df* = 3, *p* = 0.000). The Bonferroni corrected comparisons at all the time points toward *Baseline* showed a consistent and persistent improvement for all these measures (Post intervention, at 1 Month and at 3 Months, *p* < 0.05 consistently). The lack of significant main factor *Group* and *Group by Time interaction* ruled out an effect of cTBS on these parameters (Figure 4).

#### Efficiency

**Figure 5:**
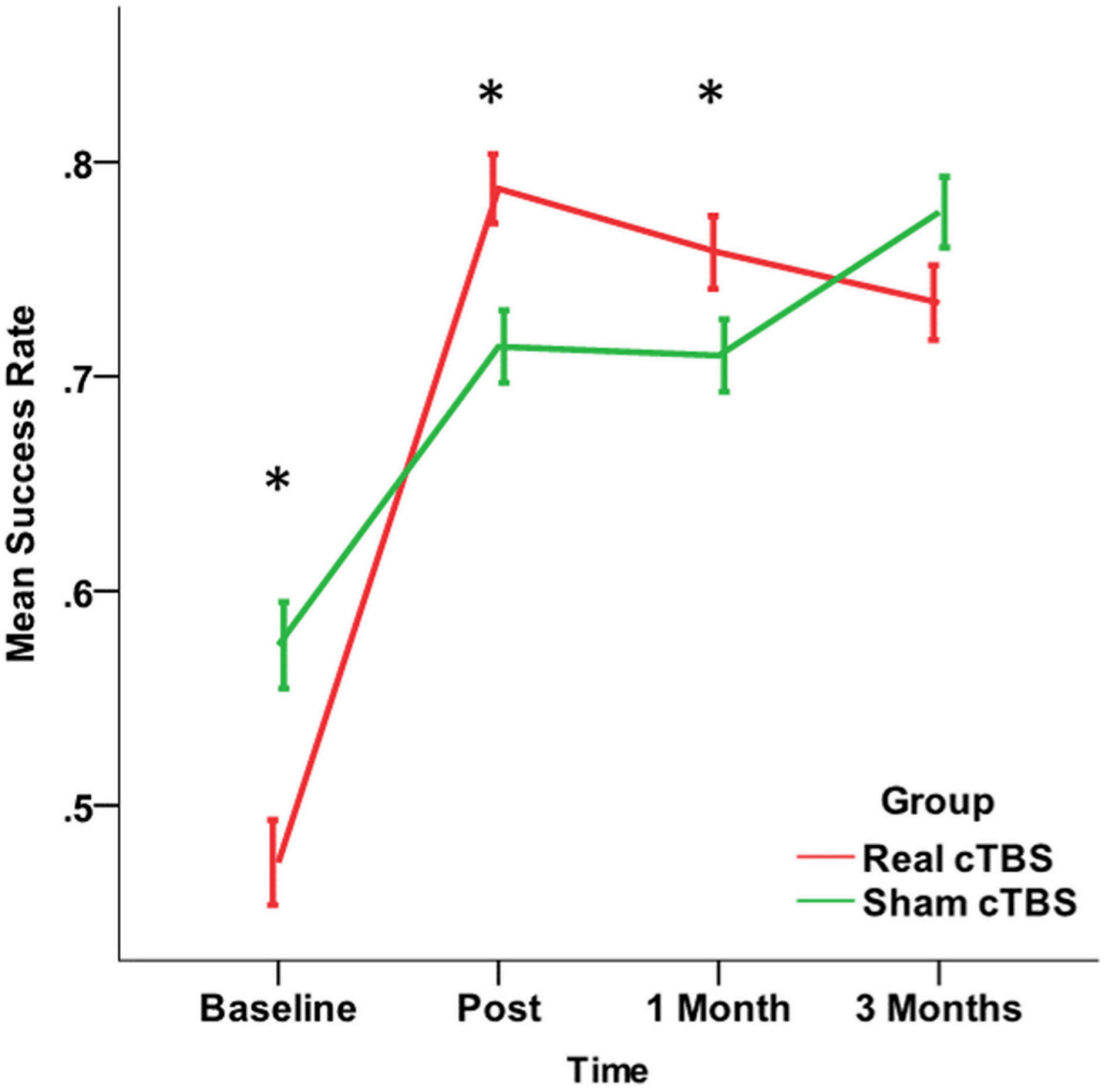
**Changes in the Success Rate, a secondary Outcome Measure broader measure of motor performance representing the percentage of times that the patient reaches the target**. The improvement in the real cTBS group was higher than in the sham group at t1 (post-treatment) and t2 (1 month). ^*^p < 0.05.

The study of the efficiency showed that cTBS over the affected hemisphere has an impact on the improvement of motor performance produced by the rehabilitation. Such effect was not unveiled by the measure of the path length (PL), for which both main Factors and interaction were not significant, but became clear at the analysis of the Success Rate. Indeed, the number of errors at *Baseline* (across groups) was 593 and decreased after the intervention, being 342 at *Post*, 364 at *1 Month*, and 313 at *3 Months*. However, the improvement was different in the two *cTBS groups* (Chi-Square = 35.576, *df* = 3, *p* = 0.000). The *Real cTBS group* showed a higher error number (337 vs. 256, Std. Residual −2.4) at *Baseline*. However, in spite of this, the study of the residuals revealed that, compared to the cTBS group, the errors were significantly more in the *sham 1* at *Post* (*Real cTBS* 136, *Sham cTBS* 206, Std. Residuals >1.9) and *1 Month* (Real cTBS 155, Sham cTBS 209, Std residuals = 1.9). Such effect was no more present at *3 Months* (3 Months; Real cTBS 170, Sham cTBS 143, Std residuals = 1.2) (Figure 5).

## Discussion

The present study shows that a robot-assisted rehabilitation protocol lasting 2 weeks produces a slight, but significant, clinical improvement in chronic stroke patients with severe upper limb motor deficits.

This study also shows that non-invasive brain stimulation delivered as cTBS over the affected hemisphere does not enhance the clinical gains from this treatment. Indeed, considering the primary outcome, there was no significant difference between real and sham-cTBS patients. The improvement in Fugl-Meyer was significant for both groups immediately after the intervention and at 1 month follow-up, while it was not significant at 3 months. At 3 months there was a slight further increase in the average scores, and the lack of significance was probably due to the high variability of the measures.

The mean change in the Fugl-Meyers score was rather limited, about 5% (3–4 points). However, this might be considered meaningful in chronic patients, especially in those with severe impairment (Lo et al., 2010). In a more general sense, a minimum increase of about five points is required to make a clinically significant difference (Page et al., 2012), but this threshold was established in patients with minimum to moderate impairment and does not fit well with our group of patients with severe impairment.

It should also be noted that, assuming that a Fugl-Meyers score difference of at least four points is of clinical interest, in order to find a significant difference between our two groups a much larger sample size might be needed (50 patients per group, Power = 80%, Type I error = 0.05). In any case, the percentage of the patients assigned to real cTBS who achieved a gain of at least five points, was slightly higher than the percentage of patients who achieved this gain in the sham group [3 out of 8 (38%) in the real group vs. 2 out of 9 (22%) in the sham group].

Interestingly, the mean improvement in the Fugl-Meyer score is comparable to what has been achieved previously with longer lasting interventions (12 weeks with 36 1-hour/day sessions of robot-assisted rehabilitative therapy in the study of Lo et al., 2010); 8 weeks with a total 24 sessions in the study by Klamroth-Marganska et al. (2014). In contrast with present findings, our previous study in chronic stroke patients with moderate upper limb deficits suggested that cTBS might enhance the gain from a late rehabilitation with a standardized protocol of physical rehabilitation (Di Lazzaro et al., 2013). One possible explanation for this discrepancy is that robot assisted rehabilitation attains a maximal benefit in patients with severe deficits and this cannot be enhanced by brain stimulation because of a ceiling effect. Another possibility, is that, as suggested by Daly et al. (2005), a more prolonged robotic therapy is needed to obtain a consistent improvement in patients with severe impairment and thus, it cannot be excluded that prolonging the association of robotic treatment and brain stimulation for a longer period might result more effective. Finally, a further possibility could arise from the fact that the affected and unaffected hemispheres seems to play a different role in mild vs. severe strokes, so that the hemisphere mainly responsible for motor recovery in severe stroke is the unaffected one (Di Pino et al., 2014b). If this is the case, in patients with severe brain damage, it may be not useful to attempt to promote ipsilesional reorganization because the manipulation of the excitability of the affected hemisphere may not produce any advantage in terms of promoting relearning from rehabilitation. Instead, in these patients, our future efforts should target the unaffected hemisphere being its role in recovery more relevant (Di Pino et al., 2014b).

It should be considered that, for safety concerns, stimulus intensity was estimated from the unaffected hemisphere, because this might be hyperexcitable (Di Lazzaro et al., 2010), it might be that this intensity was below the one needed to activate intracortical networks of the affected hemisphere. Although cTBS after effects are produced by stimulus intensities well below motor threshold (Huang et al., 2005), and although this intensity produced significant effects in our previous study in patients with less severe stroke (Di Lazzaro et al., 2013), we cannot exclude that higher intensity cTBS could produce a more pronounced effect also in patients with severe stroke.

The study of the robotic measures of motor performance (secondary outcomes) allowed us a more sensitive and accurate evaluation of the effects of robotic rehabilitation and brain stimulation on motor recovery (Pellegrino et al., 2012). These measures complement the clinical scales and show that our rehabilitation strategy achieves a significant benefit up to 3 months after the end of the treatment, confirming previous studies (Prange et al., 2006; Kwakkel et al., 2008; Lo et al., 2010). A significant improvement was achieved in multiple domains of motor control in both groups (Motion accuracy, Motion Direction, Smoothness, Speed, Movement Duration, Success Rate) with no significant difference between groups. Only the Success Rate, representing the percentage of times that the patient reaches the target, improved significantly more in the *real cTBS* group than in the *sham cTBS* one: this might suggest a mild benefit of cTBS on rehabilitation. Nevertheless, this finding should be taken extremely cautiously, since the difference between real and sham groups was not significant on the other robot-derived measures. Despite the secondary outcome measures have been analyzed in an independent fashion, we cannot rule out that the Success Rate, being a broader measure of motor performance, capitalizes the slight improvements in multiple domains of motor control, resulting statistically significant (Zollo et al., 2011a,b). However, it should also be considered that the success rate was different at baseline between the groups, this imbalance might influence the changes observed in the two groups, and this is a further reason that led to consider with caution the more pronounced improvement in success rate after real cTBS.

## Conclusions

Our study confirms that robot-assisted rehabilitative treatment produces a slight improvement years after a stroke and it shows, for the first time, that an improvement can be obtained even in patients with severe upper-limb impairment treated daily for only 10 working days. Moreover, it shows that non-invasive brain stimulation delivered as cTBS of the affected hemisphere to promote homeostatic metaplasticity, is not effective in patients with severe deficits as those enrolled in present study, while our previous study showed that this approach might be effective in patients with moderate deficits. It might be that in severe patients the unaffected hemisphere is more involved in recovery, thus, the modulation of the excitability of this hemisphere could produce positive effects. In these patients, it could also be that the facilitation of the affected hemisphere is more effective than inhibition. Also, it might be that different strategies for promoting homeostatic plasticity might produce positive effects (e.g., protocols of so-called primed stimulation in which low-frequency rTMS is preceded by a bout of high-frequency rTMS; Cassidy et al., 2015).

The implementation of non-invasive brain stimulation techniques as an additional tool to promote recovery in chronic stroke patients requires further studies in order to identify the subgroups of patients that most likely will respond to a particular intervention.

## Author contributions

VD designed the study, made the preliminary recruitment of patients and wrote the manuscript. FC was responsible of patients' recruitment and neuromodulation and revised the manuscript. GD participated to patients' recruitment, neuromodulation and revised the manuscript. GP performed statistic analysis and revised the manuscript. LF, FR, NB, MC performed the experimental sessions and the clinical evaluations. LZ, DS, EG analyzed the data collected by the robot, participated to design the robotic rehabilitative protocol, and wrote the related part of the manuscript. Sandra Miccinilli, MB and SS designed and performed the rehabilitative protocol and revised the manuscript. Stefano Milighetti revised the manuscript. All Authors approved the final version of the manuscript.

### Conflict of interest statement

The authors declare that the research was conducted in the absence of any commercial or financial relationships that could be construed as a potential conflict of interest.
